# Systematic Evaluation of Different Fresh Cow Monitoring Procedures

**DOI:** 10.3390/ani13071231

**Published:** 2023-04-01

**Authors:** Felix König, Andrew Hancock, Christian Wunderlich, Marcus Klawitter, Thomas Breuer, Anne Simoni, Karina Weimar, Marc Drillich, Michael Iwersen

**Affiliations:** 1 University Clinic for Ruminants, Clinical Unit for Herd Health Management in Ruminants, University of Veterinary Medicine, 1210 Vienna, Austria; 2 Zoetis International, D18 T3Y1 Dublin, Ireland; 3 Zoetis Germany GmbH, 10785 Berlin, Germany; 4 FFoQSI GmbH—Austrian Competence Centre for Feed and Food Quality, Safety and Innovation, Technopark 1D, 3430 Tulln, Austria

**Keywords:** dairy cow, animal welfare, animal management, precision livestock management

## Abstract

**Simple Summary:**

Metabolic and infectious diseases in dairy cows around parturition are frequent. The routine monitoring of fresh cows is recommended to detect diseases at an early stage. This, however, prevents the cows from eating and lying down, which decreases milk yield, and is time consuming for farmers, resulting in reluctant implementation on farms. Optimizing fresh cow management procedures can therefore improve efficiency and increase farmers’ acceptance of the programs. In this study, the duration of different routine examinations and treatments during fresh cow management procedures were systematically evaluated under practical conditions. In addition, different workflow strategies were investigated to determine how lock-up times could be reduced. Moreover, the proportion of examination and treatment times relative to the total headlock time was analyzed. Different examination and treatment times were found during routine fresh cow checks. Furthermore, the lock-up times differed significantly among the different strategies, as well as the proportion. This study provides information that can be used as a basis for the development of time-efficient strategies, and to minimize the impact on cows’ time budgets as well as for future herd health management strategies, including economic evaluations.

**Abstract:**

Establishing fresh cow monitoring procedures is considered beneficial for cow health, welfare, and productivity. However, they are time consuming and require the cows to be locked up, which restricts their natural behavior. In this study, different fresh cow monitoring procedures were evaluated. Two experiments were conducted to determine: (1) the duration of various examinations and treatments; (2) the time cows remain locked up in headlocks; and (3) the proportion of examination and treatment times relative to the total headlock time. In advance, standard operating procedures were established. Three veterinarians conducted the examinations and treatments based on changes in milk yield, clinical symptoms, and alarms by an accelerometer system. The headlock time was evaluated for three workflow strategies, which differed in the order of examinations and treatments. To determine the duration, cameras were installed, and the video footage was analyzed. The examinations lasted between 1 and 227 s, and the cows were locked up in headlocks between 0.01 and 1.76 h. The lock-up times differed significantly among the three strategies, as well as the proportion. This study provides information that can be used as a basis for the development of time-efficient strategies, and to minimize the impact on cows’ time budgets.

## 1. Introduction

The welfare, housing, and care of farm animals are becoming increasingly important to society [1,2]. Resting time is one indicator considered as useful regarding the well-being of dairy cows, and plays an important role in health and productivity, and accounts for part of a dairy cow’s time budget [1,3,4]. In general, the cow’s time budget can be divided into the following activities: eating, drinking, resting, standing, and perching in the alley [3,5,6]. In this context, Grant [3] pointed out that 20 to 21 h per day are required to fulfill the natural behavioral needs of a dairy cow. In addition, Cook [5] calculated that of a cow’s 24 h time budget, 2.8 h remain for milking without restricting the cows’ natural behavior. Consequently, one milking event should not take longer than one hour, assuming a milking routine with three milkings per day. Considering this short time budget, daily management protocols (e.g., examinations, treatments, lock ups in headlocks) should be carefully planned to minimize interference with the cows’ natural behavior [3,6,7,8,9]

Around parturition, 30% to 50% of dairy cows are affected by metabolic and infectious diseases, resulting in economic revenue losses, a higher risk of culling and death, and higher veterinary costs. [10,11]. These diseases are often caused by a negative energy balance, reduced feed intake, insulin resistance, and a reduced immune function [11]. On many farms, dairy cows are therefore examined several times within the first one to two weeks of lactation [12]. In this context, the intensive monitoring of fresh cows is an important factor for the success of the cow’s further lactation [11,13,14,15]. The aim of fresh cow monitoring is therefore to detect disorders at an early stage and to treat the cows if necessary [16]. The most common examinations during the fresh cow checks are the measurement of rectal temperature, rectal examination, and rumen auscultation [12]. With increasing herd size and restricted labor capacity, the amount of farmer’s and/or employee’s available time per animal decreases [17]. Furthermore, many farms have difficulties in recruiting well-trained staff to carry out these jobs.

Developing well-structured protocols for monitoring fresh cows is becoming an increasingly important management task on farms [12,18]. Many farmers check their fresh cows daily. Heuwieser et al. [19] published a survey of German farmers (*n* = 429 farms, 91% of the farms <200 dairy cows) and reported that 64% of the participants performed a daily fresh cow check. Based on the results of a survey with 45 farms in California (mean herd size 2581 cows), Espadamala et al. [12] reported that 80% of farmers carried out a daily fresh cow check, albeit at varying intensities. In contrast, Couto Serrenho et al. [20] found, in a survey in Canada (*n* = 78, herd mean of 125 ± 100 cows), that only 27% of the respondents performed a complete physical fresh cow examination. A less rigid monitoring program with partial physical examination was conducted by 64% of the participants. These daily fresh cow checks, however, are time consuming and can lead to long lock-up periods, which can severely restrict the natural behavior of the cows [3,6,21,22]. To the authors’ knowledge, there are hardly any studies on the duration of examination and treatment times that are systematically recorded in practice. Many survey-based publications are based on farmers’ perceptions of time [19,20]. 

In this study, a systematic analysis of video footage was conducted to determine the duration of various examinations, treatments, the lock-up times during the fresh cow checks as well as the proportion of examination and treatment times relative to the total headlock time. In addition, the time spent by each investigator for examinations and treatments was measured separately. The lock-up times were determined for three different workflow strategies, which differed in the order of examinations and treatments.

The objectives of this study were therefore to assess: (1) the duration of different routine examinations and treatments commonly performed in fresh cow management; (2) the time cows spend locked up in headlocks during fresh cow monitoring procedures; and (3) the proportion of the examination and treatment times in relation to the total headlock time per cow. For this, two experiments were conducted on a commercial dairy farm, involving three veterinarians.

## 2. Materials and Methods

### 2.1. Animals, Housing, and Feeding

This study was approved by the State Office of Agriculture, Food Safety, and Fisheries, Mecklenburg-Vorpommern, Germany (7221.3-2-013/21), and noted by the Ethics Committee of the University of Veterinary Medicine, Vienna. The experiments were conducted from June 2021 to August 2021 on a commercial dairy farm in Mecklenburg-Vorpommern, Germany. The farm housed approximately 1900 Holstein-Friesian dairy cows in free stall barns, with an average energy-corrected milk yield (ECM; based on 4% butterfat and 3.4% protein) of 10,301 kg per cow in 2021.

First lactating and multiparous cows were kept in different groups according to their reproductive status. The cows were milked three times a day in a 48-side-by-side parlor. Dry cows in the far-off period (i.e., from drying off at approx. 60 days before to 21 days before the expected calving date) and cows from day 10 in lactation were kept in free stall barns with concrete floors and manure scrapers in pens for up to 200 cows. The pens were equipped with cubicles bedded with a slurry separator material.

Cows entering the close-up period, i.e., from approx. 21 days before the expected calving date, were moved to a “special need” barn and kept in groups of about 150 cows. The special need barn was equipped with a 12-side-by-side parlor, in which the fresh cows were milked twice a day in the morning (05:30) and afternoon (17:30). This barn also included a compartment for diseased cows, designed to keep a focus on sick, fresh, close-up, and calving cows by the farm staff.

**Figure 1:**
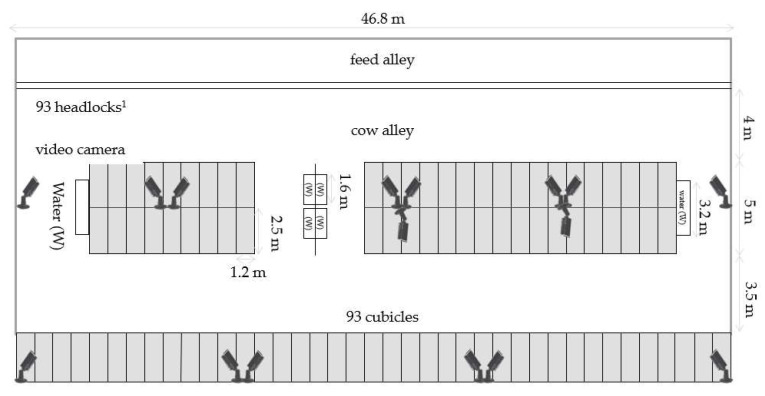
Stable plan: Illustration of the fresh cow pen with the feed alley, cubicles, and the position of the digital observation cameras. ^1^ Twist & Lock headlocks (GEA Farm Technologies GmbH, Bönen, Germany), m, meter.

As soon as cows showed signs of approaching parturition—i.e., udder edema, loosening of pelvic ligaments—they were regrouped in straw boxes with up to five other cows. After calving and colostrum harvesting, cows were moved to the fresh cow pen and remained there for the next 10 days of lactation. The fresh cow area (Figure 1) was equipped with 93 headlocks (Twist & Lock Headlocks, GEA Farm Technologies GmbH, Bönen, Germany), full concrete floors, and 93 cubicles with horse manure and chopped straw as bedding material. The feeder space per cow was 0.75 m, and the floor space per cow was 9.8 m^2^. The headlocks had an additional feature (structural tool), allowing the lock up of individual cows by a “twist and lock” system (TL) while releasing the other cows. The cows were fed a total mixed ration (TMR) ad libitum once a day in the morning (04:30), and the feed was pushed up to six times per day. The components of the TMR (% of dry matter) were grass silage (30.5%), corn silage (25.7%), forage rye silage (10.0%), corn-cob-mix and corn mix (10.2%), soybean (4.6%) and rape extraction meal (5.6%), rape seed meal (3.6%), chopped straw (2.8%), soybean hulls (1.2%), vinasse (2.8%), and minerals (3.0%). Cows had ad libitum access to water in six water troughs (3.2 m or 1.6 m per trough).

All cows were equipped with an ear-attached accelerometer SMARTBOW (SB; Smartbow/Zoetis LLC, Berlin, Germany). The data were sent in real-time and alert messages (e.g., rumination and estrus alerts) were generated using specific algorithms. Rumination alerts were influenced by rumination behavior of the individual cow, which differed from the individual behavior the days before. At the beginning of the daily examination routine in the morning, all SB alerts were screened, and the conspicuous cows were noted and subsequently examined in the fresh cow pen. 

### 2.2. Video Observation

The fresh cow pen was equipped with 16 digital observation cameras (network camera HYU-405, HYUNDAI Corporation, Seoul, Republic of Korea) at a height of approximately 3.5 m. Eight of these were directed at the feeding fence (Figure 1) and were used to evaluate the fixation, examination, and treatment times. The remaining cameras in the second row were used to monitor the cows in the holding area. The videos were stored on two network-attached NAS devices (RS3617RPxs, Synology Incorporated, New Taipei City, Taiwan) in the farm office. The client software surveillance station (version 8.2.8, Synology Incorporated, New Taipei City, Taiwan) was used to record, store, and manage video footage of cow behavior. All videos from the 16 digital observation cameras were captured simultaneously and time-synchronously by use of a time server. Mangold INTERACT (version 17.1.0.0, Mangold International, Arnstorf, Germany), a specialized software for the visual evaluation of video footage, was used to analyze the time required for the fresh cow management procedures (e.g., examinations, treatments, lock ups). For this, the start and end times of each examination and treatment time were defined in standard operating procedures (SOPs) (Table 1 and Table 2). The video footage was visually labeled by the first author (F.K.), and specific shortcuts were defined for capturing the examination, treatment, and lock-up events in Mangold INTERACT. Using a predefined and corresponding shortcut, the respective examinations and treatments were assigned a start and end time, respectively, which were taken analogously from the video.

### 2.3. Experiment 1

For study purposes, three veterinarians (F.K., A.S., K.W.) conducted the examinations and treatments in the fresh cow pen. The experience of the veterinarians was consistent, as all three had extensive experience in performing examinations and treatments of animals. At the beginning of the study, a joint but independent assessment of all examinations, based on the evaluation schemes, was performed by the three investigators. In addition, the procedures were previously trained together to ensure the homogeneity of practices.

The procedures in the study were based on the herd health management strategy that was already implemented on the farm. Accordingly, after the morning milking (05:30), the cows were locked up in headlocks, examined, and treated (every day between 07:00 and 09:00). It was randomly assigned which investigator examined and treated the cows.

Initial examinations included taking the rectal temperature, checking for retained fetal membranes (RFM), a lack of detachment of fetal membranes within 24 h postpartum, and checking the ear and skin temperatures. The criteria for a more in-depth examination after the initial check were as follows: rectal temperature ≥ 39.5 °C, RFM, signs of hypocalcemia (cold surface temperature and cold ears), deviations in the daily milk yield (i.e., ≥25% milk yield loss compared to the previous day starting with 4 DIM), and SB rumination alert. Based on these criteria, the additional examinations included: (1) estimation of rumen fill (palpation caudal to the last rib in the paralumbar fossa [23]; (2) rumen auscultation; (3) percussion; and (4) succession auscultation (if a displaced abomasum (DA) was suspected, i.e., fecal consistency scoring of 1 or 2 and/or rumen fill scoring 1 or 2 [23]); (5) evaluating fecal consistency [23]; (6) rectal examination; (7) udder examination (if the rectal temperature was ≥39.5 °C); (8) determination of the dehydration status of the eye [24]; (9) vaginal examination (if the rectal temperature was ≥39.5 °C by gloved hand, checking for injuries and vaginal discharge); (10) beta-hydroxybutyrate measurement (BHB ≥ 1.2 mmol/L) with on-site test (if ketosis were suspected (i.e., fecal consistency scoring of 4 or 5 [23])); and (11) calcium measurement (Ca ≤ 0.9 mmol/L) with on-site test (if hypocalcemia was suspected (i.e., fecal consistency scoring of 4 or 5)).

According to the clinical findings, cows were treated (Table 2) and times for treatments and applications recorded. For ketosis prophylaxis, cows received 300 mL of propylene glycol (1,2-Propandiol, Spezialfutter Neuruppin GmbH & Co. KG, Neuruppin, Germany) on days 1 and 2 of lactation. In case of a diagnosed ketosis, 300 mL of propylene glycol was administered (BHB ≥ 1.2 mmol/L and ≤1.8 mmol/L) as well as a glucose infusion (BHB > 1.8 mmol/L). If hypocalcemia was diagnosed, the cow received a calcium bolus. In case of RFM, uterine pessaries were administered to the cow. Moreover, if a rumen dysfunction (i.e., rumen fill score of ≤2 or reduced rumen contraction) was apparent, the cow received a glucose infusion or a drench. In case of fever, the cow received an anti-inflammatory treatment (intramuscularly (i.m.); subcutaneous (s.c.)). If the cows had fever for more than three days and/or metritis/mastitis, the cow received an antibiotic treatment.

### 2.4. Experiment 2

The total time each cow spent in the headlock during the fresh cow checks was recorded. To avoid bias caused by the time that was needed to bring all cows to the headlocks, recording of the headlock time started when all cows were locked up (after the morning milking). If a cow was released from the headlock, the individual lock-up time ended. To compare the lock-up times as part of the cows’ time budgets, three workflow strategies (S1, S2, and S3) were defined and performed on a set day of the week. All three strategies were carried out by two investigators. In S1, one investigator measured the rectal temperature in the entire group in a row after all cows had been locked up. Once the rectal temperature of the entire group was measured, a second investigator released all cows except for the animals that required prophylactic treatments and more in-depth examinations (individual lock up using the TL system). Both investigators examined and treated the remaining cows and documented their respective findings on a printed protocol sheet. Both investigators used the examination and treatment instruments that were placed in the feed alley. In strategy S2, only one investigator was standing in the cow alley and examined and treated the cows from behind, while a second investigator stood in the feed alley, documented all findings, and handed over all necessary treatment and examination tools. Cows were examined and treated one after the other: rectal temperature was measured, and if appropriate, further examinations were carried out and (prophylactic) treatments administered. Each cow was immediately released from the headlock after the examinations and treatments were completed. In strategy S3, one investigator measured the rectal temperature in the whole group in a row, while a second investigator checked the ears and skin temperatures from the feed alley at the same time. Then, both investigators further examined and treated the cows. All cows remained in the headlock until the treatment of the last cow was completed. Here, both investigators also documented their findings and used the examination and treatment instruments that were placed in the feed alley. The distribution of investigators standing in the cow alley or feed alley during the three different strategies was determined at the beginning of the study with a detailed protocol of duty. If both investigators examined and treated the cows, as in S2 and S3, this was randomly performed. 

### 2.5. Statistical Analysis

The results of the video analyses performed with Mangold INTERACT were exported to an Excel spreadsheet (Excel 2010, Microsoft Corporation, Remond, WA, USA). The data were analyzed with the statistical software SPSS (version 27, IBM Corporation, Armonk, NY, USA). Examinations, treatment, and lock-up times were tested for normal distribution using the Kolmogorov–Smirnov test. For comparison of the three different examination strategies (S1, S2, and S3) as well as the examination and treatment times needed by the three investigators, the Kruskal–Wallis test and analysis of variance (ANOVA) were used, respectively. Intra- and inter-rater reliabilities (documented video and examinations among the investigators) were calculated by the use of Kappa (*κ*) [25], as well as by the intraclass correlation coefficient (*r^ICC^*). The level of significance was set at *p* = 0.05. The results are presented as mean ± SD.

## 3. Results

### 3.1. Intra- and Inter-Rater Reliability

To determine the intra-rater reliability for the documented events by video analysis, the first author (F.K., Investigator 1) analyzed the video sequences of one observation day twice (i.e., 101 events in total), resulting in an agreement of event times *κ* > 0.89. The inter-rater reliability of the recorded diagnosis among the three different investigators (Inv1, Inv2, and Inv3) was determined by a joint but independent assessment of all examinations, based on the evaluation schemes (*n* = 117 cows, excluding the vaginal examination (*n* = 15), which was only carried out in clinical metritis cases) at the beginning of the study. An inter-rater agreement with *κ* ranging from 0.47 to 0.99 and *r^ICC^* = 0.97 was found (Table 3). 

### 3.2. Experiment 1

The study period compromised 60 days. Fresh cow examinations and treatments could not be carried out in a standardized way on two days (e.g., due to excessive milking times and examinations/treatments that were performed by the herdsmen in an unstandardized manner). Examinations and treatments of 58 observation days were finally analyzed, including a total of 3994 examinations and 568 treatments. 

#### 3.2.1. Duration of Examinations

In total, 3994 examinations were analyzed (Table 4). The most frequent examinations were as follows: (1) temperature measurement (*n* = 2239, 56% of all examinations); (2) succession auscultation (*n* = 325, 8.1%); (3) percussion auscultation (*n* = 329, 8.2%); and (4) rumen fill estimation (*n* = 238, 5.9%). Temperature measurement lasted a mean of 16 ± 4 s and differed among the three investigators, with a maximum difference of 0.6 ± 0.2 s (*p* < 0.01). The succession and percussion auscultation took a mean of 2 ± 1 s and 6 ± 4 s, respectively (maximum differences among the investigators of 1.2 ± 0.2 s (*p* = 0.96), and 4.3 ± 0.5 s (*p* < 0.01), respectively). The rumen fill estimation lasted a mean of 1 ± 1 s. The longest examination was the rumen auscultation, which lasted for 102 ± 33 s and differed most among the investigators, with 35.9 ± 6.3 s (*p* = 0.05). Further details are presented in Table 4 and Table 5.

#### 3.2.2. Duration of Treatments

In total, 568 treatments were analyzed (Table 6). According to the predefined (prophylactic) treatment protocols, propylene glycol was administered most frequently (*n* = 379, 66.7% of all treatments), which took a mean of 14 ± 6 s and differed among the three investigators, with a maximum difference of 2.6 ± 0.8 s (*p* = 0.05). Furthermore, i.m. and s.c. injections were frequently administered (*n* = 122, 21.4%) and took a mean of 8 ± 6 s. The longest treatment step was due to infusions (*n* = 29, 5%), which lasted a mean of 482 ± 127 s. Further details are presented in Table 6 and Table 7. 

### 3.3. Experiment 2

The study period compromised 60 days. Twenty-two observation days were lost for the evaluation of the headlock times. On these days, the procedures and strategies could not be carried out in a standardized way because the farm staff selected cows to be moved in other pens. This happened on various days throughout the study period and led to prolonged lock-up times. Headlock times of 44 observation days were finally analyzed, including a total of 1848 headlock times.

#### Lock-Up Times

**Figure 2:**
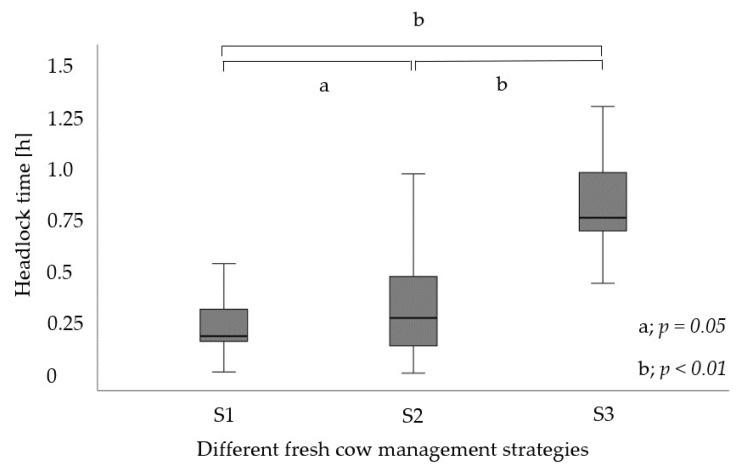
Distribution of headlock times per cow (h) for the different fresh cow management strategies (S1–S3). Outliers are not shown in the boxplot. Significant differences are represented as follows: a; *p* = 0.05, b; *p* < 0.01. hours, h.

In total, 1848 headlock times of cows observed during 44 days were used for statistical analyses, divided among the three fresh cow management strategies: S1 (*n* = 562, 30.4%), S2 (*n* = 674, 36.4%), S3 (*n* = 612, 33.1%). During the daily fresh cow checks, 40 ± 6.9 cows were housed in the fresh cow pen (minimum: 26 cows, maximum: 56 cows). Across the three different strategies, examinations and treatments were evenly distributed. Figure 2 shows the differences in the lock-up times of the cows among the strategies. By mean, the cows were locked up in S1 for 0.29 ± 0.28 h, whereas in S2, they were locked up for 0.33 ± 0.26 h. In S3, the cows were locked up for 0.81 ± 0.23 h. Fifty percent of the cows left the headlocks after 0.18 h in S1, after 0.26 h in S2, and after 0.75 h in S3. Differences were found between S1 and S2 (*p* = 0.05) as well as between S1 and S3 and between S2 and S3 (*p* < 0.01). In S1, 5.0% (*n* = 29) of the cows remained in headlocks for more than 1 h, in S2, 4.0% (*n* = 27), and in S3, 13.7% (*n* = 84), with a maximum of 1.76 h.

The proportion of examination and treatment times in relation to the total headlock time per cow was 3.6 ± 3.6%, 8.6 ± 16.7%, and 1.5 ± 2.6% in S1, S2, and S3, respectively. Differences were found between S1 and S2 (*p* = 0.04) as well as between S1 and S3 and between S2 and S3 (*p* < 0.01).

## 4. Discussion

Guterbock [18] and Espadamala et al. [12] highlighted the importance of well-structured fresh cow management protocols during the transition period to identify and treat sick cows at an early stage. For example, infections and inflammations can be detected by measuring the rectal temperature. As part of regular monitoring of fresh cows, between 36% and 89% of farmers reported that rectal temperature was measured daily [12,19,26]. In this study and due to the SOP that all cows had to be measured at the initial examination, the measurement of the rectal temperature was also the most frequent procedure (56% of all conducted examinations). Measuring the rectal temperature took a mean of 16 ± 4 s (*n* = 2239). Among the investigators, a maximum difference of 0.6 ± 0.2 s (*p* < 0.01) was detected, which can be neglected from a practical point of view. Guterbock [18] assumed that the measurement of rectal temperature as well as its documentation took 60 s per cow. It should be noted that in our study, the documentation was limited to marking the cow with color marks as the end point of the temperature measurement. Guterbock [18] did not describe an exact endpoint but noted that the color-coding of febrile and healthy cows allows for the quickest possible identification. In addition, Guterbock [18] did not specify which thermometer was used to measure the rectal temperature. In this study, a digital thermometer was used, which generally allows for a faster measurement than analog thermometers.

Furthermore, cows are often screened for DA as this is a frequent and costly disease in early lactation [16,27,28]. Typical indicators for DA are poor performance and reduced rumen motility. Rumen motility should be determined with a stethoscope or by hand in the paralumbar fossa, as described by Guterbock [18]. In this study, succession and percussion auscultation took a mean of 2 ± 1 s (*n* = 325) and 6 ± 4 s (*n* = 329), respectively. In total, the combined examination of succession and percussion auscultation took a mean of 8 s, which was even shorter than measuring the rectal temperature. Espadamala et al. [12] described that 67% of the surveyed farms had stethoscopes for rumen diagnostics, but only 20% of them used them when DA was suspected. It should be noted that the time–benefit ratio for succession and percussion auscultation is favorable, but it requires trained personnel to diagnose DA.

Moreover, one of the first and most common examinations of fresh cows is a visual examination to obtain a first impression of the general attitude of the cow [17,18]. Part of this is the estimation of rumen fill caudal to the last rib in the paralumbar fossa. To determine this, the five-point scoring system of Zaaijer et al. [23] is often used. In this experiment, the estimation of rumen fill took a mean of 1 ± 1 s (*n* = 238), with a maximum difference among the investigators of 1.2 ± 1.2 s (*p* < 0.01), which can be neglected from a practical point of view. Heuwieser et al. [19] and Espadamala et al. [12] described that 35% and 11%, respectively, of surveyed dairy farmers estimated the rumen fill. Burfeind et al. [29], however, described that the results are highly subjective in their repeatability, and considerable diurnal variations can occur. The diurnal variation should therefore be considered when evaluating this parameter. In this study, however, the inter-rater reliability for the rumen filling score showed a great inter-rater agreement (*κ* = 0.97, *n* = 114), because the cows were always examined at a similar time of day. 

Examination and treatment times that are systematically recorded in practice have not, to our knowledge, been obtained so far and the survey-based publications rely on farmers’ perceptions of time [19,20]. For example, Espadamala et al. [12] described that on 45 farms, the fresh cow checks took 0.08 to 4 h per herd or 1 to 46 s (mean 16 s) per cow. Furthermore, we also measured the time that was needed for cow treatments. The application of propylene glycol was the most frequently performed treatment (66.7% of all treatments); it took a mean of 14 ± 6 s. As the oral administration of propylene glycol is one of the most effective options for the prevention and treatment of ketosis [30], our data provide valuable information on how much time is spent on this. 

It can be speculated that the times needed for examinations and treatments differed between investigators depending on, e.g., their professional experience and individual thoroughness. In future studies, these differences should be determined with a larger number of investigators as well as in different farm settings. Nevertheless, the systematic numbers obtained in this study can be used as a benchmark for, e.g., the economic evaluation of fresh cow management protocols.

An important goal in fresh cow management is to keep cows in headlocks for less than one hour to minimize disruption to their natural behavior [22]. In our study, 5.1% (S1), 4.0% (S2), and 13.7% (S3) of the cows remained in the headlocks for more than one hour. The results of S1 and S2 are similar to those obtained from other surveys, where 8.9% and 3.9% of the fresh cows remained in headlocks for more than one hour [12,20]. Kerwin et al. [26] described that even 13.9% were locked up between one and three hours in headlocks during routine fresh cow checks, which is comparable to S3 in our study.

A structural tool used in our study was the TL system, which allows for keeping individual cows in headlocks while releasing others. In accordance with the fresh cow management described by Aalseth [31], one person was in the feed alley and another person was working behind the cows while they were examined one after the other. All three strategies were performed by two investigators. This procedure was also described by Espadamala et al. [12], who reported that in large herds (as in our study farm), fresh cow checks were usually performed by two or more employees. Similarly, the strategies described in this study cannot be carried out by a single person. This is a factor to be taken into account in practical implementation, which can be limiting, especially when there is a lack of personnel. A targeted training of personnel, possibly for simpler examinations or documentation activities, may be useful for this purpose. The strategy S1 (using the TL system to restrain cows requiring treatments and/or in-depth examinations), with a mean headlock time of 0.29 ± 0.28 h, proved to be the most time-efficient one compared with S2 and S3. Strategy 3, where all cows remained in the headlock until the treatment of the last cow was completed, proved to be the least time-efficient one as the cows remained in the headlocks the longest. Only a small difference was found between S1 and S2. In S2, where one investigator examined and treated the cows from the cow alley, while a second investigator worked in the feed alley and documented all findings, the cows remained in the headlocks for a mean of 2 min longer than in S1, but the variation was significantly greater compared to S1. Strategy 3 was performed according to the farm’s standard procedure, but the cows remained in the headlocks the longest, i.e., 0.81 ± 0.23 h. In summary, the headlock time per cow can be significantly reduced by the order of examinations and treatments. 

The proportion of examination and treatment times in relation to the total time in the headlock per cow was further determined. This ratio reflects the efficiency of the respective strategies to minimize headlock times. It should be mentioned that only examination and treatment times were recorded, while the time for the documentation of findings and preparation of treatments were not considered. Strategy 3 showed the smallest proportion of examinations and treatments in relation to the total headlock time per cow, at 1.5 ± 2.6%. In other words, 98.5% of the time a cow spent in the headlocks was not used for examinations and treatments. Out of the three strategies, strategy 3 can be considered the most ineffective. By providing systematically recorded examination and treatment times from three different veterinarians, this study can contribute to the evaluation of already established management strategies on farms or the development of new strategies that have the smallest possible impact on the time budget of the cows. Follow-up studies should investigate to what extent different lock-up times of cows in headlocks influence their time budgets for other activities such as feed intake or lying and thus, limiting their natural behavior. Furthermore, the association of the fixation time and the performance of the cows, such as an increase in daily milk yield and reproductive performance but also on the recovery time in case of diseases should be investigated. Based on this, optimal management strategies for fresh cows can be developed, considering the needs of the cows, the number of available staff, and other economic aspects. 

## 5. Conclusions

The duration of different examinations and treatments in fresh cow management was presented, as well as significant differences in the examination and treatment times of three different veterinarians. These, however, can be neglected from a practical point of view. Of greater importance, significant differences in the lock-up times of the cows in the headlocks were determined for different fresh cow management strategies. Particular attention should be paid to the proportion of examination and treatment times in relation to the total lock-up time of the cows. Moreover, the results of this study can support future developments for optimized management strategies, considering the cows’ needs as well as available working capacity and other economic factors. 

## Figures and Tables

**Table 1:** Definition of start and end points for various examination steps, including the necessary diagnostic instruments and tools.

Item	Definition		Instruments and Diagnostic Tools
Examinations	Start	End	
Measurement of rectal temperature	The thermometer is inserted rectally	The cow is marked with a red/yellow marker	Veterinary digital thermometer VET 12, TFA Dostmann GmbH & Co. KG, Wertheim, Germany
Estimation of rumen fill	The paralumbar fossa is palpated, caudal to the last rib	The hand is taken back	
Rumen auscultation	A functional stethoscope is put on	Fingers touch the abdomen to perform the percussion	Stethoscope Prof. Dr. Götze, Herberholz GmbH & Co. KG, Solingen, Germany
Percussion auscultation	Fingers touch the abdomen to perform the percussion	Formation of a fist (start point of succession)	Stethoscope Prof. Dr. Götze, Herberholz GmbH & Co. KG, Solingen, Germany
Succession auscultation	The succession starts by the formation of a fist	The functional stethoscope is taken off	Stethoscope Prof. Dr. Götze, Herberholz GmbH & Co. KG, Solingen, Germany
Feces examination	The rectal glove is put on (shoulder protection over the head)	The rectal glove is removed (pull the shoulder protector over the head again)	Manuplast Vet shoulder, B. Braun Vet Care GmbH, Tuttlingen, Germany
Rectal examination	The rectal glove is put on (shoulder protection over the head)	The rectal glove is removed (pull the shoulder protector over the head again)	Manuplast Vet shoulder, B. Braun Vet Care GmbH, Tuttlingen, Germany
Udder examination	Investigator squats down	Investigator stands again	
Dehydration	The investigator’s hand moves to the cow’s eye	The hand is withdrawn again	
Vaginal examination	The rectal glove is put on (shoulder protection over the head)	The rectal glove is removed (pull the shoulder protector over the head again)	Manuplast Vet shoulder, B. Braun Vet Care GmbH, Tuttlingen, Germany
Beta-hydroxybutyrate/Calcium measurement	The blood tube is opened	The result is presented on the device	Freestyle Precision ß-Ketone, Abbott Diabetes Care Ltd., Oxon, United Kingdom; CaQuicktest LAQUAtwin-Ca-11-C, QUIDEE GmbH, Homberg, Germany

**Table 2:** Definition of start and end points for various animal treatments, including the necessary materials.

Item	Definition		Material
Treatments	Start	End	
Infusion	Halter is put on	Halter is taken off	Infusion set, Hauptner&Herberholz, Solingen, Germany; Glucose, 500 mL, B.Braun Vet Care GmbH, Tuttlingen, Germany
Injection i.m. ^1^; s.c. ^2^	The investigator touches the cow (skin fold, push up the tail)	The investigator steps away from the cow	Bovivet 14G 2.1×60 mm, Jørgen Kruuse A/S, Langeskov, Denmark
Uterine pessary	Pessary is removed from the packaging	The rectal glove is removed (pull the shoulder protector over the head again)	Tetra-Bol 2000 mg, cp-pharma, Burgdorf, Germany
Oral drench	The investigator inserts the drench tube into the cow’s rumen	The drench tube is pulled out of the cow	SELEKT Pump-Drencher, Trademark of Nimrod Veterinary Products Ltd., Gloucestershire, United Kingdom
Propylene glycol	The investigator approaches the cow with a propylene glycol drench gun	The investigator steps away from the cow	Drench 300 mL, Wahl GmbH, Dietmannsried, Germany
Calcium bolus	The investigator approaches the cow with a calcium bolus applicator	The investigator steps away from the cow	BOVIKALC, Boehringer Ingelheim, Denmark

^1^ intramuscularly; ^2^ subcutaneous.

**Table 3:** Inter-rater reliability: three investigators (Inv1–Inv3) were compared with each other in various examinations, Cohen’s Kappa (κ), intraclass correlation coefficient (rICC).

Examination	Number of Events	*κ* ^1^	*r^ICC^* ^1^
Measurement of rectal temperature	112		0.97
Estimation of rumen fill	114	0.97	
Rumen auscultation	117	0.99	
Succession and percussion auscultation	117	0.99	
Feces examination	115	0.89	
Rectal examination	117	0.47	
Udder examination	117	0.99	
Dehydration	117	0.99	
Vaginal examination	15	0.99	

^1^ *p* < 0.001 for Cohen’s Kappa and for intraclass correlation coefficients.

**Table 4:** Duration of the examinations (obtained from all three investigators).

Examination			Duration (Seconds)					
	*n* ^1^	Median	IQR ^2^	Mean	SD ^3^	Min	Max	90th P ^5^
Measurement of rectal temperature	2239	15	4	16	4	5	42	21
Estimation of rumen fill	238	1	1	1	1	1	13	4
Percussion auscultation	329	6	5	6	4	1	32	11
Succession auscultation	325	2	2	2	1	1	9	4
Fecal examination	87	26	15	29	13	12	78	47
Udder examination	204	16	9	18	12	4	102	28
Vaginal examination	74	59	30	63	27	18	135	106
Rumen auscultation	149	101	40	102	33	33	227	143
Rectal examination	148	37	20	39	13	15	74	60
Dehydration	140	2	2	2	1	1	14	5
BHB ^4^ measurement	31	28	4	28	2	24	36	33
Calcium measurement	30	17	12	20	6	14	32	30

^1^ number of examinations, ^2^ Interquartile range, ^3^ Standard deviation, ^4^ beta-hydroxybutyrate, ^5^ percentile.

**Table 5:** The duration of examinations presented for each of the three investigators (Inv) and differences among the investigators (mean ± SD).

Examination	Inv ^1^		Duration (Seconds)						Differences among Inv ^1^ (Seconds)		
		*n* ^2^	Median	IQR ^3^	Mean	SD ^4^	Min	Max	1 vs. 2	2 vs. 3	1 vs. 3
Measurement of rectal temperature	1	831	15	4	16	3	6	39	< 0.1 ± 0.2 (*p* = 1.00)	0.6 ± 0.2 (*p* < 0.01)	0.5 ± 0.2 (*p* = 0.02)
	2	847	15	5	16	3	9	42			
	3	561	15	4	15	3	10	41			
Estimation of rumen fill	1	84	1	1	1	0	1	9	0.2 ± 0.2 (*p* = 1.00)	0.9 ± 0.2 (*p* < 0.01)	1.2 ± 1.2 (*p* < 0.01)
	2	79	1	1	1	1	1	9			
	3	75	2	3	2	1	1	13			
Percussion auscultation	1	103	9	3	9	3	3	22	4.3 ± 0.5 (*p* < 0.01)	1.4 ± 0.5 (*p* = 0.02)	2.9 ± 0.5 (*p* < 0.01)
	2	136	4	3	4	3	2	28			
	3	90	5	3	6	4	1	32			
Succession auscultation	1	103	2	1	2	0	1	7	0.6 ± 0.1 (*p* < 0.01)	0.4 ± 0.2 (*p* = 0.03)	1.2 ± 0.2 (*p* = 0.96)
	2	127	2	1	1	1	1	6			
	3	95	2	2	2	1	1	9			
Fecal examination	1	38	21	8	22	7	12	49	8.3 ± 3.0 (*p* = 0.03)	6.8 ± 3.2 (*p* = 0.11)	15.1 ± 2.8 (*p* < 0.01)
	2	22	28	10	31	14	14	78			
	3	27	36	15	37	12	18	66			
Udder examination	1	62	14	6	15	5	7	33	1.0 ± 2.0 (*p* = 1.00)	7.8 ± 2.0 (*p* < 0.01)	8.9 ± 2.1 (*p* < 0.01)
	2	75	14	8	16	12	4	102			
	3	67	19	12	24	15	9	95			
Vaginal examination	1	29	66	39	72	25	37	132	26.2 ± 7.5 (*p* < 0.01)	20.3 ± 7.7 (*p* < 0.01)	6.0 ± 6.9 (*p* = 1.00)
	2	19	37	17	46	27	18	132			
	3	26	58	21	66	24	35	135			
Rumen auscultation	1	43	107	52	115	38	65	227	4.2 ± 5.9 (*p* = 1.00)	31.7 ± 5.8 (*p* < 0.01)	35.9 ± 6.3 (*p* = 0.05)
	2	61	110	34	111	26	57	183			
	3	45	74	43	79	24	33	119			
Rectal examination	1	42	28	9	30	7	15	51	9.7 ± 2.3 (*p* < 0.01)	6.8 ± 2.3 (*p* = 0.01)	16.5 ± 2.5 (*p* < 0.01)
	2	62	38	16	39	12	22	73			
	3	44	44	23	46	13	21	74			
Dehydration	1	48	2	2	2	1	1	6	0.4 ± 0.3 (*p* = 0.58)	0.9 ± 0.4 (*p* = 0.02)	0.5 ± 0.3 (*p* = 0.52)
	2	49	2	3	2	2	1	14			
	3	43	2	2	2	0	1	4			
BHB ^5^ measurement	1	10	28	5	29	3	26	36	2.9 ± 1.0 (*p* = 0.03)	2.4 ± 1.0 (*p* = 0.10)	0.5 ± 1.1 (*p* = 1.00)
	2	11	26	2	26	1	24	29			
	3	10	29	2	29	2	26	33			
Calcium measurement	1	10	27	13	23	6	14	31	5.1 ± 2.5 (*p* = 0.17)	0.8 ± 2.6 (*p* = 1.00)	5.9 ± 2.6 (*p* = 0.09)
	2	10	18	5	18	5	14	32			
	3	10	16	5	17	5	14	31			

^1^ Investigator, ^2^ number of examinations, ^3^ interquartile range, ^4^ standard deviation, ^5^ beta-hydroxybutyrate.

**Table 6:** Duration of the individual treatments (from all three investigators).

Treatment/ Administration			Duration (Seconds)					
	*n* ^1^	Median	IQR ^2^	Mean	SD ^3^	Min	Max	90th P ^4^
Infusion	29	453	155	482	127	316	844	684
Injection i.m. ^5^; s.c. ^6^	122	8	4	8	6	1	52	15
Uterine pessary	15	62	46	76	32	38	153	132
Oral drench	2	186		180	38	159	212	
Propylene glycol	379	13	6	14	6	5	51	22
Calcium bolus	21	16	5	17	8	8	48	25

^1^ number of treatments/administrations, ^2^ interquartile range, ^3^ standard deviation, ^4^ percentile, ^5^ intramuscularly, ^6^ subcutaneous.

**Table 7:** Duration of examinations presented for each of the three investigators (Inv) and differences among the investigators (mean ± SD).

Treatment/ Administration	Inv ^2^		Duration (Seconds)						Difference among Inv ^2^ (Seconds)		
		*n* ^1^	Median	IQR ^3^	Mean	SD ^4^	Min	Max	1 vs. 2	2 vs. 3	1 vs. 3
Infusion	1	13	464	142	487	139	341	844	49 ± 60 (*p* = 1.00)	70 ± 65 (*p* = 0.85)	21 ± 55 (*p* = 1.00)
	2	7	361	246	438	127	316	627			
	3	9	457	160	509	112	338	726			
Injection i.m. ^5^; s.c. ^6^	1	28	7	3	9	5	1	26	0.8 ± 1.4 (*p* = 1.00)	0.3 ± 1.2 (*p* = 1.00)	0.5 ± 1.5 (*p* = 1.00)
	2	49	8	6	8	4	1	20			
	3	45	7	5	8	7	1	52			
Uterine pessary	1	6	59	54	77	39	50	153	11 ± 27 (*p* = 1.00)	17 ± 27 (*p* = 1.00)	5 ± 18 (*p* = 1.00)
	2	2	88		88	42	58	118			
	3	7	65	44	71	26	38	112			
Oral drench	1										
	2										
	3	2	186		186	38	159	212			
Propylene glycol	1	211	12	5	13	5	5	51	2 ± 0.9 (*p* = 0.03)	0.2 ± 1.0 (*p* = 1.00)	2.6 ± 0.8 (*p* = 0.05)
	2	63	13	10	15	8	7	41			
	3	105	14	7	16	7	7	51			
Calcium bolus	1	9	17	7	16	4	11	26	3 ± 5 (*p* = 1.00)	6 ± 5 (*p* = 0.62)	3 ± 4 (*p* = 1.00)
	2	4	13	9	13	4	8	18			
	3	8	15	5	19	12	13	48			

^1^ number of treatments/administrations, ^2^ investigator, ^3^ interquartile range, ^4^ standard deviation, ^5^ intramuscularly, ^6^ subcutaneous.

## Data Availability

Not applicable.
